# A study on the mental health and body weight of Chinese college students in the context of COVID-19: implications for educational practice

**DOI:** 10.3389/fpubh.2025.1687465

**Published:** 2026-02-12

**Authors:** Shuzhen Ma, Jingzhi Zhang, Yanqi Xu, Jinliang Liu

**Affiliations:** 1 College of Materials Science and Engineering, Guilin University of Technology, Guilin, China; 2 Department of Rehabilitation Therapy, Nanjing Mingzhou Rehabilitation Hospital, Nanjing, China

**Keywords:** Chinese, college students, COVID-19, overweight/obese, psychological state

## Abstract

The COVID-19 pandemic has significantly impacted the physical and mental health of college students. However, there is a lack of investigation into the psychological state and overweight/obesity prevalence among Chinese college students post-COVID-19. This study aims to provide evidence-based insights to guide mental health and weight management education for university students in China. Data from 4,484 Chinese college students (3,565 men and 919 women) were collected in 2022 and 2023, including symptom checklist 90 (SCL-90) scale results and body mass index (BMI) measurements. Frequency analysis, independent and paired-samples *t*-tests, and logistic regression were conducted using SPSS software. The results indicate that 12.4% of students were overweight/obese in 2023, with a higher prevalence among men (14.6%). Psychological abnormalities were observed in 8.7% of students, with a higher proportion in women (9.5%). Overweight/obese students showed no significant differences in 10 psychological indicators. Logistic regression analysis revealed that for every 1-point increase in somatization score, the odds of students being overweight/obese increased by 100.1% (men: 94.1%, women: 244.6%). Hostility scores were also associated with higher odds of overweight/obesity (overall increase of 78.1%; men: 69.5%, women: 272.2%). In contrast, a 1-point increase in phobic anxiety score was associated with lower odds of being overweight/obese (overall reduction of 67.5%; men: 54.0%, women: 298.8%). This study found that COVID-19 had a minimal impact on the body weight of Chinese college students but significantly affected their mental health. The obesity rate among female students has increased, with key psychological issues contributing to this trend, including obsessive-compulsive disorder, interpersonal sensitivity, and depression. Specifically, somatization and hostility were positively associated with the odds of being overweight/obese, while phobic anxiety was negatively associated, with these effects being more pronounced in female students.

## Background

Over the last three decades, there has been a concerning global rise in the frequency of overweight and obesity among children and adolescents (1, 2), which is especially obvious in China. The rapid growth of China’s economy has led to a significant increase in the proportion of overweight and obese people (3–5). During the past 40 years, the overall obesity rate in China has increased dramatically, making it one of the highest worldwide (6). The issue of obesity in China has gradually evolved into a significant social problem. This situation is prevalent among students, who suffer from severe physical and mental health problems and thus pose a potential threat to social development (7). Therefore, in China, the body mass index (BMI) is measured annually to assess the weight of university students. The test results are expressed as BMI (kg/m^2^) values (8). In clinical practice, BMI is the most commonly used metric for diagnosing obesity and for providing suggestions for weight management and control (9, 10).

According to clinical psychological studies, obese children and adolescents often experience psychological symptoms, such as poor self-esteem, low self-confidence, decreased happiness, and reduced life satisfaction (11). Compared with their normal-weight peers, overweight/obese adolescents are more prone to exhibit risky behaviors, such as verbal, physical, and relational victimization (12). Global concern has also been raised regarding college students’ mental health (13). Severe mental illness and obesity both have detrimental effects on quality of life and are associated with higher rates of morbidity, mortality, and disability (14). Because they are in a unique transitional state from adolescence to adulthood, college students are particularly vulnerable to mental health issues (15). China experienced the COVID-19 pandemic from December 2019 to December 2022. The effects of the COVID-19 pandemic on the health of children and adolescents represent a significant but sometimes overlooked concern (16). During the COVID-19 pandemic, China implemented strict control measures, including lockdowns, quarantines, and online education (17–20). Although these policies effectively curbed the spread of the virus, they had substantial impacts on the mental and physical health of college students. Studies have shown that social constraints and school closures resulting from long-term home isolation may make children and adolescents feel more isolated, which may lead to a rise in mental health or overweight/obese issues (21, 22). However, few studies have examined whether the lifting of COVID-19 restrictions in China has been beneficial for the mental or physical health of adolescents.

Students with mental health issues may find it more difficult to focus, experience reduced motivation, and have difficulty communicating with others, all of which can have a negative impact on their academic performance (23). Importantly, serious mental illnesses (such as depression) are among the primary causes of suicide and impose a significant burden on individuals, families, and society (23). Previous research has shown that approximately 20.9% of Chinese college students have considered suicide and that over 30% of them experience depression (24, 25). The Symptom Checklist-90 (SCL-90) scale has demonstrated good reliability in assessing mental health and has become one of the main tools used to measure psychological symptoms. It has proven to be reliable (26). The SCL-90 scale is often used in China to examine the mental health status of college students. In the SCL-90 scale, respondents rate the 90 questionnaire items on a 5-point distress scale, where 0 represents “not at all” and 4 represents “very much.” (27) The checklist includes the following nine main symptom dimensions: somatization, obsessive-compulsive, interpersonal sensitivity, depression, anxiety, hostility, phobic anxiety, paranoid ideation, and psychoticism (28). Owing to social restrictions and school closures, children and adolescents in a prolonged state of home isolation may become more lonely, which may lead to an increase in mental health issues (21).

For a long time, researchers have been very interested in the interrelationship between psychopathology and obesity (29). In previous research, numerous studies aimed to investigate whether obesity leads to mental health problems, including anxiety and depression (30). Numerous findings suggest that obese individuals are more likely to experience sadness and anxiety than those with a normal weight (31–35). In addition to its confirmed physiological effects, obesity is also associated with a wide range of medical diseases, making it a complex condition (36). Reports from mental health facilities indicate that many individuals with obesity experience neurotic or characterological disturbances related to issues of reliance, growth, and nurturing (37). By contrast, there is currently a lack of research examining how mental states may contribute to the development of obesity or overweight.

Furthermore, numerous studies have explored the impact of various factors, including self-isolation, on obesity during the COVID-19 pandemic (38–42). At the same time, a large number of literature has also investigated the effects of the COVID-19 pandemic on the mental health of college students (43, 44). Among them, Kim et al. (45) specifically studied the changes in college students’ mental health before and during the pandemic. However, the existing research generally lacks longitudinal comparisons between the pandemic and post-pandemic periods, and there are also relatively limited comprehensive studies on the interaction between mental health and obesity.

This study aims to fill gaps in the existing literature and conduct a detailed analysis of the relationship between obesity and mental health among Chinese college students. Specifically, it examines the changes in these variables before and after the lifting of restrictions during the COVID-19 pandemic. Additionally, the study also explores the sex differences in overweight/obesity and mental health conditions, with a particular focus on the psychological changes experienced by students after the pandemic. This research provides valuable insights into the impact of the pandemic on students’ mental health and helps to understand the interrelationship between obesity and mental health in this context.

## Hypothesis

Based on existing theories and research, the following hypotheses are proposed:

*H1*: Owing to social isolation and college closures, it is hypothesized that the mental health of college students would significantly deteriorate during the COVID-19 pandemic (21, 46, 47).

*H2*: During the pandemic, due to home quarantine and reduced physical activity, the body weight of college students would increase, leading to a rise in the prevalence of overweight and obesity (46, 48, 49).

*H3*: There is a close relationship between mental health status and overweight or obesity. This hypothesis is based on existing findings that psychological stress and social isolation are associated with weight gain (50, 51).

Therefore, this study aims to examine the dual impact of the COVID-19 pandemic on the mental health and body weight of college students and to explore the relationship between changes in mental health and body weight.

Based on the above hypotheses, this study evaluates the body weight and mental health of Chinese college students using BMI tests and the SCL-90 scale. The BMI tests are used to measure students’ body-weight status, while the SCL-90 scale is used to assess various dimensions of mental health (52, 53). Independent sample *t*-tests and logistic regression analyses are used to compare the differences in psychological states between normal-weight students and overweight/obese students and to evaluate the impact of mental health indicators on body-weight status (54, 55). Furthermore, by comparing data from 2022 to 2023, the study will investigate changes in the mental health and body weight of college students before and after the pandemic. It is expected that during the pandemic, there would be a close relationship between students’ mental health status and body weight. This study aims to provide empirical evidence for understanding the impact of the COVID-19 pandemic on the mental health and body weight of college students, and to provide references for formulating relevant intervention measures (21, 56, 57).

## Method

### Participants

The study covered the Northern and Northeastern, Northwestern, and Southwestern regions of China and involved eight universities. The study was approved by the Ethics Committee of Guilin University of Technology in accordance with the Declaration of Helsinki (58). Participants were undergraduate students from the first to fourth years, with ages typically ranging from 18 to 23. In each university, 150 students were selected from each academic year, yielding a total of 4,800 participants initially recruited. Students were voluntarily recruited from various departments within each university. To ensure the representativeness of the sample, a random sampling technique was employed, covering different majors and reflecting the diversity of academic disciplines.

In order to control potential interfering factors and reduce the differences in socioeconomic status, lifestyle, and health conditions among college students, we selected public universities with the smallest differences in these factors. Through the assistance of university teachers in sports and mental health, we completed the recruitment process and provided academic credits as an incentive to the participants. This approach was approved by the ethics committee because it has educational significance, thereby motivating students to actively participate. Based on the order of registration by grade, the top 150 applicants from each grade were selected, and informed consent was obtained orally from the participants. A total of 4,800 participants underwent examinations related to their physical and mental health. After excluding participants with incomplete data and questionnaire errors, the data of 4,484 students were included in the study, including 3,565 men and 919 women.

**Figure 1:**
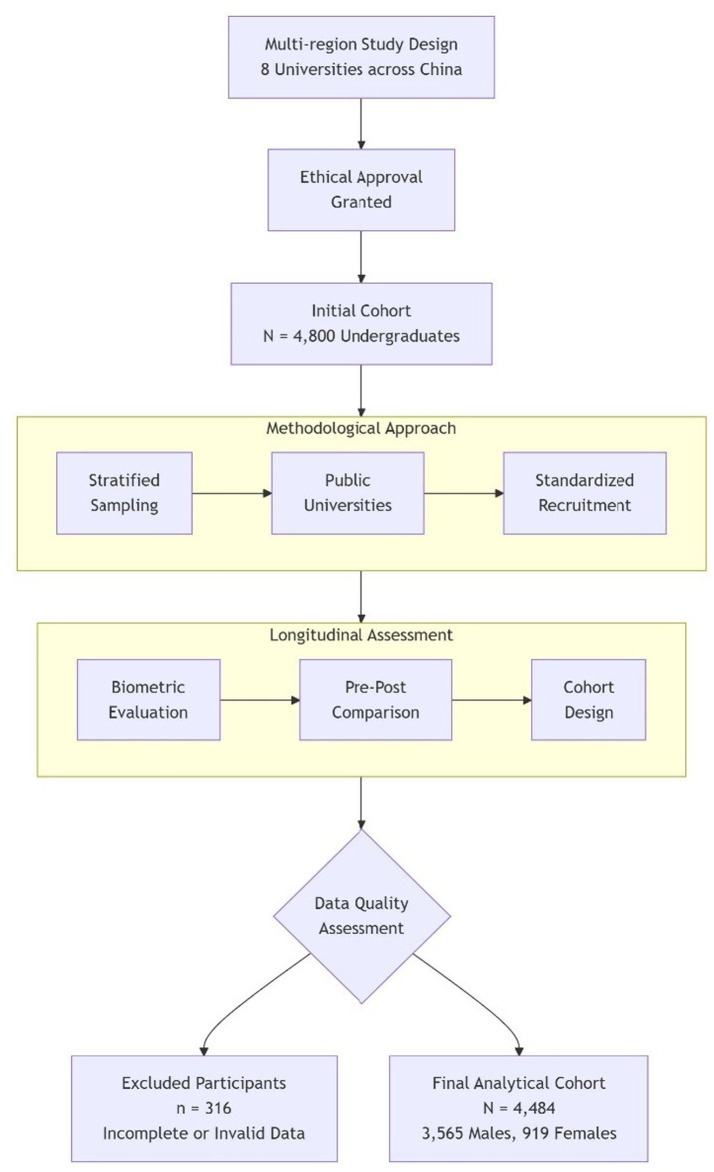
Study process of the impact of COVID-19 on the physical and mental health of Chinese college students.

The data were collected between January 2022 and August 2023, covering two periods before and after the COVID-19 lockdown. The same group of students was surveyed in both periods, enabling a direct comparison of their physical and mental conditions before and after China lifted the restrictions on COVID-19. This design ensures that the comparison reflects changes in the same individual rather than independent annual samples. The comparison group is defined as follows: (1) students who were surveyed before the restrictions were lifted, and (2) students who were surveyed after the lockdown measures were lifted. This study ensures that data were collected from students of different backgrounds, covering various disciplines and economic backgrounds, to comprehensively assess the impact of the epidemic measures on students’ physical and mental health. The detailed study protocol is outlined in Figure 1.

### Assessment

This study employed questionnaire surveys and BMI tests for evaluation. During the BMI test, the testing indicators were conducted in accordance with the Chinese Ministry of Education’s National Physical Health Standards (NPHS), and the height and weight of the students were measured. As part of the graduation requirements, Chinese college students are required to undergo a height and weight assessment once a year. All students who were eligible and consented to participate completed the assessment, which was administered by the physical education teachers at the universities. According to the NPHSS standards, the BMI score is calculated by dividing body weight by the square of height.

A widely used psychiatric questionnaire is the SCL-90 (27). Previous studies have confirmed that this questionnaire has high reliability (59). The SCL-90 psychological scale consists of 90 items and is divided into 10 symptom dimensions: somatization, obsessive-compulsive, interpersonal sensitivity, depression, anxiety, hostility, phobic anxiety, paranoid ideation, psychoticism, and others. Teachers from eight universities distributed this questionnaire to students *via* their mobile phones, and the students were instructed to complete it accurately and independently. The response rate of this questionnaire was 100%, and the validity response rate was 94%.

### Variable table

The unit of BMI score is kg/m^2^, and it is calculated by dividing body weight by the square of height. The evaluation indicators for men and women are different (Table 1) (60). It is worth noting that underweight was defined as a BMI score ≤17.8 for men and ≤17.1 for women.

**Table 1:** BMI variable table.

BMI (kg/m^2^)		Indicator connotation
Male	Female	
17.9 ~ 23.9	17.2 ~ 23.9	Normal
≤17.8	≤17.1	Underweight
24.0 ~ 27.9	24.0 ~ 27.9	Overweight
≥28.0	≥28.0	Obese

BMI, body mass index.

The SCL-90 scale has a total of 90 items, divided into 10 factors (Table 2) (61). Factor scores were calculated based on the standard scoring method for the SCL-90 scale. Each item adopts a five-level scoring system, with the following definitions:

**Table 2:** SCL-90 variable table.

Type	Factor	Factor score	Abnormal	Normal
Somatization	1, 4, 12, 27, 40, 42, 48, 49, 52, 53, 56, 58	S/N	1–2	>2
Obsessive-compulsive	3, 9, 10, 28, 38, 45, 46, 51, 55, 65			
Interpersonal sensitivity	6, 21, 34, 36, 37, 41, 61, 69, 73			
Depression	5, 14, 15, 20, 22, 26, 29, 30, 31, 32, 54, 71, 79			
Anxiety	2, 17, 23, 33, 39, 57, 72, 78, 80, 86			
Hostility	11, 24, 63, 67, 74, 81			
Phobic anxiety	13, 25, 47, 50, 70, 75, 82			
Paranoid ideation	8, 18, 43, 68, 76, 83			
Psychoticism	7, 16, 35, 62, 77, 84, 85, 87, 88, 90			
Others	19, 44, 59, 60, 64, 66, 89			

S, sum of the scores of each factor item; *N*, number of items in each factor.

1 = None: Feeling that there is no symptom (problem) in this item—score 1.

2 = Very mild: The symptom is felt but has no actual impact, or the impact is slight—score 2.

3 = Moderate: The symptom is consciously present and has a certain impact—score 3.

4 = Severe: Feeling that this symptom often occurs and has a considerable impact—score 4.

5 = Very severe: The symptom is very frequent and intense and has a serious impact—score 5.

Factor score = sum of the scores of each factor item/number of items in each factor.

### Data analysis

This study assessed SCL-90 scale results and BMI in a cohort of college students in China. Frequency analysis was performed on the total scores of both the BMI and SCL-90 scale tests for the entire sample (60). Additionally, frequency analysis was conducted for the BMI level tests and SCL-90 scale test results based on sex differences.

Statistical analysis was conducted using statistical product and service solutions (SPSS) software, including independent sample *t*-tests, paired-samples *t*-tests, and logistic regression analysis. Specifically, independent-samples *t*-tests were used to compare psychological state differences between overweight/obese and normal-weight students, with a significance level set at 0.05. Logistic regression analysis was employed to assess the impact of psychological indicator scores on students’ weight status. In this logistic regression analysis, the dependent variable (weight status) was dichotomized into normal weight (coded as 1) and overweight/obesity (coded as 2). Owing to potential multicollinearity among the predictors, multicollinearity diagnostics, such as the variance inflation factor (VIF), were conducted to assess stability. The binary logistic regression model was used to explore the impact of psychological test scores, including somatization, obsessive-compulsive, interpersonal sensitivity, depression, anxiety, hostility, phobic anxiety, paranoid ideation, psychoticism, and others, on students’ weight status. To further compare differences between BMI and psychological test scores in 2022 and 2023, paired-samples *t*-tests were performed.

## Results

### BMI level test results

In 2023, a total of 4,484 college students in China participated in body mass index (BMI) and psychological tests, including 3,565 male students and 919 female students. The results of the total score of the BMI classification are presented in Table 3. From Table 3, it can be seen that a total of 447 students were classified as obese, accounting for 10.0% of the total number of students; a total of 109 students were classified as overweight, accounting for 2.4% of the total; there are 3,343 students classified as having normal weight, accounting for 74.5% of the total students, representing the highest proportion; there are 585 students classified as underweight, accounting for 13.0% of the total students. The proportions of male samples who were overweight/obese or underweight were higher than those of female samples.

**Table 3:** Frequency and percentage of Chinese college students in different weight categories (*n* = 4,484).

Weight category	Overall (*n* = 4,484)		Boys (*n* = 3,565)		Girls (*n* = 919)	
	Frequency	Percent (%)	Frequency	Percent (%)	Frequency	Percent (%)
Obesity	447	10.0	416	11.7	31	3.4
Overweight	109	2.4	102	2.9	7	0.8
Normal weight	3,343	74.5	2,553	71.6	790	86.0
Underweight	585	13.0	494	13.9	91	9.9
In total	4,484	100.0	3,565	100.0	919	100.0

### SCL-90 scale test results

Our previous study shows the frequency distribution of SCL-90 scale test results (60). From our previous study, it can be seen that, across the 10 dimensions of the SCL-90 scale, the number of students with normal psychological test results was greater than that with abnormal results, and the proportion of students with abnormal results accounted for 8.7% of the total sample. The proportions of abnormal psychological test results in the female and male samples were 9.5% and 8.5%, respectively. In the overall sample, the three most prevalent abnormal dimensions were obsessive-compulsive symptoms (13.4%), interpersonal sensitivity (9.3%), and depression (8.3%), while somatization accounted for the lowest proportion (4.6%). In the male sample, the top three proportions were obsessive-compulsive symptoms (13.0%), interpersonal sensitivity (9.3%), and depression (7.8%), with somatization again accounting for the lowest proportion (4.5%). In the female sample, the top three proportions were obsessive-compulsive symptoms (14.8%), interpersonal sensitivity (9.5%), and depression (10.3%), while somatization accounted for the lowest proportion (5.0%).

### Differences in psychological tests between Normal weight and overweight/obesity

Supplemenary Table S1 presents differences in psychological test results between individuals with normal weight and overweight/obesity. In the overall sample, no differences were observed between the two groups in the psychological indicators of somatization, obsessive-compulsive, interpersonal sensitivity, depression, anxiety, hostility, phobic anxiety, paranoid ideation, psychoticism, and others (*p* > 0.05).

In the male sample, no differences were observed between the two groups in psychological indicators of somatization, obsessive-compulsive, interpersonal sensitivity, depression, anxiety, hostility, phobic anxiety, paranoid ideation, psychoticism, and others (*p* > 0.05).

In the female sample, no differences were observed between the two groups in psychological indicators of somatization, obsessive-compulsive, interpersonal sensitivity, depression, anxiety, hostility, phobic anxiety, paranoid ideation, psychoticism, and others (*p* > 0.05).

### Logistic regression analysis

In this study, because the dependent variable (weight state) was divided into normal weight (coded as 1) and overweight/obesity (coded as 2), a binary logistic regression model was used to examine the associations between psychological test indicator scores and students’ weight state. As shown in Table 4, four factors—somatization, hostility, phobic anxiety, and others—were significant predictors in the overall sample. Specifically, higher somatization and hostility scores increased the odds of overweight/obesity, whereas higher scores in phobic anxiety and others were associated with decreased odds.

**Table 4:** Results of psychological test logistic regression analysis between normal weight and overweight/obesity (*n* = 3,899).

	Overall (*n* = 3,899)				Boys (*n* = 3,071)				Girls (*n* = 828)			
	B	*p*	OR	95% CI	B	*p*	OR	95% CI	B	*p*	OR	95% CI
Somatization	1.001	0.000	2.722	1.634–4.535	0.941	0.001	2.562	1.478–4.443	2.466	0.017	11.776	1.563–88.708
Obsessive-compulsive	0.162	0.396	1.176	0.809–1.711	0.254	0.204	1.290	0.871–1.909	−0.723	0.350	0.485	0.107–2.209
Interpersonal sensitivity	0.412	0.111	1.510	0.909–2.506	0.240	0.377	1.271	0.747–2.163	1.200	0.283	3.319	0.371–29.692
Depression	−0.288	0.287	0.750	0.441–1.274	−0.063	0.827	0.939	0.533–1.652	−0.861	0.399	0.423	0.057–3.126
Anxiety	−0.393	0.231	0.675	0.354–1.285	−0.314	0.361	0.730	0.372–1.434	0.660	0.585	1.935	0.181–20.705
Hostility	0.781	0.001	2.183	1.350–3.531	0.695	0.006	2.004	1.218–3.296	2.722	0.010	15.210	1.927–120.069
Phobic anxiety	−0.675	0.010	0.509	0.305–0.850	−0.540	0.046	0.583	0.343–0.991	−1.521	0.181	0.218	0.024–2.031
Paranoid ideation	0.061	0.826	1.062	0.619–1.823	−0.106	0.711	0.899	0.513–1.576	1.144	0.360	3.140	0.271–36.348
Psychoticism	−0.382	0.230	0.683	0.366–1.274	−0.457	0.177	0.633	0.326–1.230	−2.988	0.024	0.050	0.004–0.674
Others	−0.555	0.015	0.574	0.367–0.897	−0.510	0.029	0.600	0.379–0.950	−2.786	0.023	0.062	0.006–0.680
Constant	−1.941	0.000	0.144		−1.773	0.000	0.170		−2.295	0.009	0.101	

B represents the non-standardized coefficient, P represents the significance, OR represents the odds ratio, and 95% CI represents the 95% confidence interval.

Sex-stratified models revealed similar patterns. For men, somatization and hostility increased the odds of overweight/obesity, while phobic anxiety and others reduced the odds. For women, somatization and hostility again increased the odds, whereas psychoticism and others reduced the odds. The magnitude of psychological effects was generally stronger in women. However, these results should be interpreted cautiously due to potential multicollinearity and the absence of multiple-comparison corrections. Future studies should report VIF values, employ continuous BMI regression models, and apply adjustment methods such as Bonferroni or false discovery rate (FDR) correction to enhance robustness.

### Paired-samples *t*-test

To further compare differences between BMI scores and psychological test scores in 2022 and 2023, this study used paired-samples *t*-tests.

1. Paired-samples *t*-test of BMI scores.

As shown in Supplementary Table S2, the average BMI score of students in 2022 was 20.4543, which was slightly lower than that in 2023 (20.5309). To determine whether this difference was statistically significant, a paired-samples *t*-test was conducted. The results are presented in Supplementary Table S3. Supplementary Table S3 shows that the *p*-value of the *t*-test statistic was 0.221, indicating that at the 5% significance level, there was no statistically significant difference between the average BMI scores in 2022 and 2023.

1. Paired-samples *t*-test of psychological test scores.

Table 5 presents the descriptive statistics of psychological test scores in 2022 and 2023. As shown in Table 5, the average scores of all 10 psychological indicators in 2022 were higher than those in 2023, indicating an overall improvement in students’ psychological status in 2023. From Table 6, it can be further concluded that the significance *p*-values of the *t*-test statistics are all less than 0.05, indicating that at the significance level of 5%, there is a significant difference between the average scores of these 10 indicators of psychological state in 2022 and their corresponding average scores in 2023.

**Table 5:** Descriptive statistics of psychological test scores in 2022 and 2023.

		Mean	*N*	Std. Deviation	Std. Error Mean
Pair 1	Somatization 2022	1.1918	4,484	0.36701	0.00548
	Somatization 2023	1.1656	4,484	0.36051	0.00538
Pair 2	Obsessive-compulsive 2022	1.5388	4,484	0.60356	0.00901
	Obsessive-compulsive 2023	1.3937	4,484	0.54640	0.00816
Pair 3	Interpersonal sensitivity 2022	1.3936	4,484	0.53626	0.00801
	Interpersonal sensitivity 2023	1.2836	4,484	0.46941	0.00701
Pair 4	Depression 2022	1.3440	4,484	0.51357	0.00767
	Depression 2023	1.2601	4,484	0.46702	0.00697
Pair 5	Anxiety 2022	1.2818	4,484	0.44960	0.00671
	Anxiety 2023	1.2130	4,484	0.41541	0.00620
Pair 6	Hostility 2022	1.2558	4,484	0.42676	0.00637
	Hostility 2023	1.2039	4,484	0.40627	0.00607
Pair 7	Phobic anxiety 2022	1.2229	4,484	0.41218	0.00616
	Phobic anxiety 2023	1.1795	4,484	0.38988	0.00582
Pair 8	Paranoid ideation 2022	1.2564	4,484	0.43676	0.00652
	Paranoid ideation 2023	1.1883	4,484	0.39542	0.00591
Pair 9	Psychoticism 2022	1.2691	4,484	0.44200	0.00660
	Psychoticism 2023	1.2008	4,484	0.40408	0.00603
Pair 10	Others 2022	1.3145	4,484	0.48037	0.00717
	Others 2023	1.2397	4,484	0.43882	0.00655

*N*, sample size; Std. Deviation, standard deviation; Std. Error Mean, standard error of the mean.

**Table 6:** Paired-samples *t*-test of psychological test scores in 2022 and 2023.

		Paired differences					t	df	Sig. (2-tailed)
		Mean	Std. Deviation	Std. Error Mean	95% CI of the difference				
					Lower	Upper			
Pair 1	Somatization 2022–2023	0.02624	0.51494	0.00769	0.01116	0.04132	3.412	4,483	0.001
Pair 2	Obsessive-compulsive 2022–2023	0.14509	0.80641	0.01204	0.12148	0.16870	12.048	4,483	0.000
Pair 3	Interpersonal sensitivity 2022–2023	0.11003	0.71042	0.01061	0.08923	0.13083	10.371	4,483	0.000
Pair 4	Depression 2022–2023	0.08393	0.69501	0.01038	0.06358	0.10428	8.086	4,483	0.000
Pair 5	Anxiety 2022–2023	0.06887	0.60973	0.00911	0.05102	0.08672	7.563	4,483	0.000
Pair 6	Hostility 2022–2023	0.05195	0.58792	0.00878	0.03473	0.06916	5.917	4,483	0.000
Pair 7	Phobic anxiety 2022–2023	0.04335	0.56937	0.00850	0.02668	0.06002	5.099	4,483	0.000
Pair 8	Paranoid ideation 2022–2023	0.06809	0.58839	0.00879	0.05087	0.08532	7.750	4,483	0.000
Pair 9	Psychoticism 2022–2023	0.06829	0.59622	0.00890	0.05083	0.08574	7.670	4,483	0.000
Pair 10	Others 2022–2023	0.07480	0.64940	0.00970	0.05579	0.09382	7.713	4,483	0.000

*t*, t-statistic; df, degrees of freedom; Sig. (2-tailed), *p*-value; Mean, mean difference; Std. Deviation, standard deviation; Std. Error Mean, standard error.

## Discussion

The BMI test results indicate that although COVID-19 had little impact on the weight of Chinese college students, among those who are overweight or obese and those who are underweight, the proportion of men is higher than that of women. A study on overweight/obesity among 11,673 Chinese college students in 2018 found that the overweight/obesity rate was 9.5%, with 13.9% for men and 6.1% for women, indicating that the overweight/obesity rate of men is higher than that of women (62–64). It is worth noting that overweight/obesity rates have risen since 2018, particularly among female students (65, 66). The increase in overweight and obesity rates among women may be partly explained by several factors. From a physiological perspective, the basal metabolic rate of women is usually lower than that of men, which means that under the same diet and exercise levels, women are more likely to accumulate fat. In addition, female students may not participate in physical exercise as frequently as their male counterparts, leading to differences in energy consumption and subsequently affecting weight control. Although the prevalence of overweight/obesity is currently higher among male students, female students may be more physiologically susceptible to weight gain due to lower basal metabolic rates and lower physical activity levels. This distinction reflects differences between observed prevalence and underlying biological mechanisms. Therefore, to address the problem of overweight/obesity among students, measures focusing on nutrition and physical exercise should be implemented (67).

The SCL-90 results indicate that a minority of Chinese college students exhibited psychological distress both before and after the pandemic, with women showing slightly higher abnormal rates than men. Mental health symptoms were mainly related to obsessive-compulsive tendencies, interpersonal sensitivity, and depression, consistent with previous findings among Chinese university students (68, 69). These patterns may be linked to intensified academic and social competition associated with rapid socioeconomic development in China (69), and prior research shows that female students continue to face comparatively disadvantaged psychological conditions despite improvements among men over the past decades (70, 71).

Body-weight perception also interacts with mental health. Adolescents who accurately recognize their weight status exhibit fewer physical discomfort symptoms but show higher levels of social withdrawal and anxiety than those misperceiving their weight (72), highlighting the dual role of body perception in shaping emotional wellbeing. Mechanistically, somatization and hostility—often associated with dysregulated stress responses—may contribute to emotional eating, metabolic dysregulation, and central fat accumulation (73, 74). These mechanisms may partly explain why psychological factors are more strongly associated with weight status in female students, who often experience greater social pressure and body-image concern.

Consistent with prior evidence linking psychological wellbeing and obesity (75–78), our regression models quantified these relationships using a large sample. Somatization and hostility significantly increased the odds of overweight/obesity, while phobic anxiety and the “others” dimension (representing sleep- and diet-related symptoms) were associated with reduced odds. These associations were more pronounced among women. Previous studies suggest that dietary regulation can alleviate somatization (79–82), and anxiety may alter dietary intake patterns (83, 84), offering potential explanations for the directionally mixed effects observed in this study. The negative association between the “others” factor and overweight/obesity may relate to the role of sleep and dietary irregularities in metabolic health (85, 86). Evidence remains limited on sex differences in psychological mechanisms underlying weight change, suggesting a need for future research to examine sex-specific pathways linking emotional distress, coping behaviors, and body-weight shifts.

In a paired-samples *t*-test of the average BMI results of Chinese college students in 2023 and 2022, no significant difference was found. However, paired-samples *t*-tests of the mean scores across the 10 SCL-90 factors showed that the psychological state of Chinese college students in 2023 had significantly improved compared with 2022. These findings indicate that the COVID-19 pandemic had a negligible impact on body weight among Chinese college students but substantially affected their mental health, which improved significantly after the pandemic. This conclusion was based on comparisons of mental health scores before and after the lifting of lockdown measures, suggesting that the pandemic’s impact on mental health was more pronounced than on physical health. The rapid spread of the novel coronavirus and its associated mortality risk may have exacerbated pre-existing mental health conditions and made public health personnel more susceptible to psychological distress. Numerous studies have shown that disasters often trigger significant psychological impacts (87). For example, within 6 months after the Wenchuan earthquake, the incidence of suicidal thoughts reached its peak (88). This difference may be attributed to the fact that the COVID-19 pandemic lasted longer than the Wenchuan earthquake, while its acute psychological impact was relatively shorter.

Therefore, the hypotheses H1 and H3 in this study were supported, while hypothesis H2 was not. A study on the impact of the COVID-19 pandemic on the psychology of college students proposed four public mental health crisis intervention strategies: organizing psychological support groups, coordinating communication to eliminate uncertainties, providing hotline counseling services, and screening and counseling confirmed patients (89). In mental crisis intervention, prevention and early intervention are considered key factors in minimizing the impact of potential severe mental health conditions, but early intervention in youth mental health is a goal that has not yet been fully achieved (90). Although progress has been made in the area of mental disorders, largely due to the successful application of the high-risk mental state concept (91), this concept has not been widely explored in the context of common mental disorders such as depression, anxiety, substance abuse, and eating disorders (92). To meet the challenges of early intervention in youth mental health, it is necessary to promote multidisciplinary collaboration among different professionals and to redesign preventive and early intervention services for young people by enhancing and integrating primary care services (93, 94). Additionally, future research should focus on accumulating empirical data and evaluating the effectiveness of these interventions through large-scale longitudinal studies. Only through continuous research and practice can effective early intervention in youth mental health be achieved.

### Limitations

The main strength of this study lies in its comprehensive empirical conclusions drawn from the investigation and correlation analysis of Chinese college students’ psychological states and overweight/obesity before and after the pandemic. However, the study has certain limitations:

1. Owing to the use of non-random sampling, the study sought to adhere to principles of equality, inclusiveness, and openness and included a relatively large sample size. However, voluntary participation and unequal sex distributions across academic disciplines led to discrepancies in the number of male and female participants. Additionally, as the study employed a cross-sectional design, it cannot establish causal relationships between psychological factors and obesity, limiting conclusions regarding cause-and-effect dynamics.
2. Important confounding variables, such as diet, physical activity, socioeconomic status, and access to mental health resources, were not directly measured in this study. Future research should consider controlling for these factors through multivariable analysis to better understand their potential influence on the relationship between obesity and mental health (23).
3. Owing to the large dataset and the number of results presented, this study did not include graphical or other visualization tools to support data interpretation.
4. The categorical nature of the “weight-status” variable may oversimplify the data, and future analyses should consider continuous BMI-based regression models to enhance robustness.

### Future research directions

Future research should address the limitations of this study to gain a better understanding of the psychological and physical health of college students. First, ensuring sample representativeness is necessary to eliminate potential biases. Additional confounding variables that may affect the results should be included, such as family background, economic status, lifestyle habits, and access to mental health resources. Longitudinal studies should be conducted to track the long-term impacts of events such as the COVID-19 pandemic on student health. The geographical scope of future research should be expanded to include universities from diverse regions and socioeconomic backgrounds, thereby enhancing generalizability. Finally, the integration of qualitative methods, such as interviews or focus groups, may provide richer and more nuanced insights.

## Conclusion

This study found that COVID-19 had a minimal impact on the body weight of Chinese college students but significantly affected their mental health. The obesity rate among female students has increased, with key psychological issues contributing to this trend, including obsessive-compulsive disorder, interpersonal sensitivity, and depression. Specifically, somatization and hostility were positively associated with the odds of being overweight/obese, while phobic anxiety was negatively associated, with these effects being more pronounced in female students. The study underscores the significant influence of psychological factors on weight status and suggests the need for sex-specific interventions.

## Data Availability

The original contributions presented in the study are included in the article/Supplementary material, further inquiries can be directed to the corresponding author.

## Glossary

SCL: symptom checklist
BMI: body mass index
NPHS: National Physical Health Standards
Somatization: Somatization refers to the manifestation of psychological distress through physical symptoms without an identifiable medical cause. These symptoms may include pain, fatigue, and gastrointestinal discomfort.
Obsessive-Compulsive: Obsessive-compulsive disorder is characterized by persistent, intrusive thoughts (obsessions) and repetitive behaviors or mental acts (compulsions) that an individual feels compelled to perform to alleviate anxiety or prevent a feared outcome.
Interpersonal Sensitivity: Interpersonal sensitivity involves heightened awareness and responsiveness to perceived social rejection, criticism, or negative evaluation by others, often resulting in feelings of inadequacy and social anxiety.
Depression: Depression is a mood disorder marked by persistent sadness, loss of interest or pleasure in activities, feelings of hopelessness, and low self-esteem. It may also present with physical symptoms such as changes in appetite, sleep disturbances, and fatigue.
Anxiety: Anxiety refers to excessive and prolonged worry and fear that interfere with daily functioning. It is often accompanied by physical symptoms such as palpitations, sweating, trembling, and gastrointestinal distress.
Hostility: Hostility is characterized by anger and antagonism toward others, manifesting as resentment, aggression, and a desire to cause harm or discomfort.
Phobic Anxiety: Phobic anxiety is an intense, irrational fear of specific objects, activities, or situations, leading to avoidance behavior that is typically disproportionate to the actual threat.
Paranoid Ideation: Paranoid ideation involves pervasive mistrust and suspicion of others’ intentions, often including beliefs that others are plotting against or intending harm.
Psychoticism: Psychoticism encompasses a range of severe psychological symptoms, including hallucinations, delusions, disorganized thinking, and behaviors detached from reality, often associated with conditions like schizophrenia.
