# Supplementary material for: A study on the mental health and body weight of Chinese college students in the context of COVID-19: implications for educational practice
Source: Front Public Health. 2026 Feb 12;13:1687465. doi: 10.3389/fpubh.2025.1687465 (PMC12935936; doi:10.3389/fpubh.2025.1687465)
Supplement: Supplementary file 1 [file Table_1.docx]

**Supplementary Materials**

**A Study on the Mental Health and Body Weight of Chinese College Students in the Context of COVID-19**

Shuzhen Ma,^1^ Yanqi Xu,^2,^ * Jinliang Liu,^1^

^1^ College of Public Administration, Guilin University of Technology, Guilin 541004, China

^2^ College of Materials Science and Engineering, Guilin University of Technology, Guilin 541004, China

* Corresponding author

E-mail: xuyanqi@glut.edu.cn

**Table S1.** Differences of psychological test between normal weight and overweight/obesity (n=3899)

|  | Overall (n=3899) | | | | Boys (n=3071) | | | | Girls (n=828) | | | |
| --- | --- | --- | --- | --- | --- | --- | --- | --- | --- | --- | --- | --- |
|  | Normal weight | Overweight/Obesity | *p* value | 95% CI | Normal weight | Overweight/Obesity | *p* value | 95% CI | Normal weight | Overweight/Obesity | *p* value | 95% CI |
| Somatization | 1.1224±0.2780 | 1.1423±0.3334 | 0.184 | (-0.049, 0.009) | 1.1186±0.2761 | 1.1383±0.3285 | 0.202 | (-0.050, 0.011) | 1.1348±0.2840 | 1.1966±0.3952 | 0.347 | (-0.193, 0.069) |
| Obsessive-compulsive | 1.3911±0.5420 | 1.384±0.5371 | 0.773 | (-0.042, 0.056) | 1.3807±0.5420 | 1.3873±0.5401 | 0.802 | (-0.058, 0.045) | 1.4248±0.5411 | 1.3395±0.4989 | 0.311 | (-0.083, 0.253) |
| Interpersonal sensitivity | 1.2795±0.4671 | 1.2753±0.4471 | 0.845 | (-0.038, 0.046) | 1.2772±0.4718 | 1.2763±0.4489 | 0.967 | (-0.043, 0.045) | 1.287±0.4518 | 1.2629±0.4277 | 0.737 | (-0.120, 0.168) |
| Depression | 1.2598±0.4690 | 1.2383±0.4214 | 0.273 | (-0.017, 0.060) | 1.2462±0.4589 | 1.2374±0.4198 | 0.686 | (-0.034, 0.052) | 1.3038±0.4977 | 1.2505±0.4494 | 0.481 | (-0.098, 0.205) |
| Anxiety | 1.2119±0.4160 | 1.1937±0.3765 | 0.333 | (-0.019, 0.055) | 1.2031±0.4177 | 1.1923±0.3789 | 0.585 | (-0.028, 0.050) | 1.2404±0.4092 | 1.2132±0.3473 | 0.642 | (-0.090, 0.145) |
| Hostility | 1.2009±0.3976 | 1.2151±0.4290 | 0.440 | (-0.050, 0.022) | 1.1961±0.4001 | 1.2121±0.4294 | 0.411 | (-0.054, 0.022) | 1.2165±0.3891 | 1.2555±0.4270 | 0.583 | (-0.182, 0.104) |
| Phobic anxiety | 1.1802±0.3848 | 1.1517±0.3684 | 0.103 | (-0.006, 0.063) | 1.1734±0.3802 | 1.1512±0.3735 | 0.226 | (-0.014, 0.058) | 1.2024±0.3986 | 1.1579±0.2926 | 0.374 | (-0.055, 0.144) |
| Paranoid ideation | 1.1863±0.3894 | 1.1808±0.3958 | 0.757 | (-0.030, 0.041) | 1.1873±0.3938 | 1.1798±0.3991 | 0.695 | (-0.030, 0.045) | 1.1832±0.3749 | 1.1939±0.3522 | 0.855 | (-0.129, 0.108) |
| Psychoticism | 1.2008±0.4018 | 1.180±0.3843 | 0.257 | (-0.015, 0.057) | 1.2019±0.4070 | 1.1844±0.3914 | 0.369 | (-0.021, 0.056) | 1.1972±0.3849 | 1.1211±0.2673 | 0.101 | (-0.015, 0.168) |
| Others | 1.2417±0.4433 | 1.2125±0.4021 | 0.119 | (-0.008, 0.066) | 1.2378±0.4496 | 1.2154±0.4090 | 0.263 | (-0.017, 0.062) | 1.2542±0.4225 | 1.1732±0.2938 | 0.112 | (-0.020, 0.182) |

**Note:** ^a^ means there is significant difference at 0.01 level.

**Table S2.** The Descriptive Statistic of BMI Scores in 2022 and 2023

|  | | Mean | N | Std. Deviation | Std. Error Mean |
| --- | --- | --- | --- | --- | --- |
| Pair 1 | BMI 2022 | 20.4543 | 4484 | 2.95035 | 0.04406 |
|  | BMI 2023 | 20.5309 | 4484 | 3.05139 | 0.04557 |

**Note:** N, Sample size; Std. Deviation, Standard deviation; Std. Error Mean, Standard error of the mean.

**Table S3.** Paired-samples Test of BMI Scores in 2022 and 2023

|  | | Paired Differences | | | | | t | df | Sig. (2-tailed) |
| --- | --- | --- | --- | --- | --- | --- | --- | --- | --- |
|  |  | Mean | Std. Deviation | Std. Error Mean | 95% CI of the Difference | |  |  |  |
|  |  |  |  |  | Lower | Upper |  |  |  |
| Pair 1 | BMI 2022 – BMI 2023 | -.07656 | 4.18499 | 0.06250 | -.19909 | 0.04596 | -1.225 | 4483 | 0.221 |

**Note:** t, t-statistic; df, Degrees of freedom.
